# The expression of p73 is increased in lung cancer, independent of *p53* gene alteration

**DOI:** 10.1038/sj.bjc.6690572

**Published:** 1999-07

**Authors:** Y Tokuchi, T Hashimoto, Y Kobayashi, M Hayashi, K Nishida, S Hayashi, K Imai, K Nakachi, Y Ishikawa, K Nakagawa, Y Kawakami, E Tsuchiya

**Affiliations:** Saitama Cancer Center Research Institute, 818 Komuro, Ina, Saitama, 362-0806, Japan; Department of Clinical Pathology, Saitama Cancer Center Hospital, 818 Kumuro, Ina, Saitama, 362-0806, Japan; Departments of Pathology and Respiratory Surgery, Cancer Institute, 1-37-1 Kami-Ikebukuro, Toshimaku, Tokyo 170-8455, Japan; First Department of Medicine, School of Medicine, Hokkaido University, Sapporo, 060-8636, Japan

**Keywords:** lung cancer, *p73*, 1p36, *p53*, tumour suppressor gene

## Abstract

*p73* gene, a new *p53* homologue, has been identified: it supposedly acts as tumour suppressor gene in neuroblastoma. To clarify whether *p73* might be involved in lung carcinogenesis, we examined *p73* expression in resected lung cancer and paired normal lung in 60 cases using semi-quantitative reverse transcription-polymerase chain reaction (RT-PCR). We also examined *p73* gene status in three representative cases using Southern blot, and *p53* gene alteration in 49 cases using PCR-single-strand conformation polymorphism (PCR-SSCP) and direct sequence. In 87% of the cases (52/60) *p73* expression in tumour was more than twice as high as that in paired normal lung tissues, and the difference between *p73* expression in tumour and normal lung tissue was significant (*P* < 0.0001). However, Southern blot analysis revealed that none of the cases showed *p73* gene amplification. Compared with clinicopathological characteristics, *p73* expression correlates significantly with histological differences and age of patient, independently (*P* < 0.05). Concerning *p53* gene status, 43% (21/49) showed *p53* gene alteration, but there was no correlation between *p73* overexpression and *p53* gene alteration. Our results suggest that need for further functional analysis of the role of *p73* in lung carcinogenesis. © 1999 Cancer Research Campaign


*British Journal of Cancer* (1999) **80**(10), 1623–1629
© 1999 Cancer Research Campaign
Article no. bjoc.1999.0572

### The expression of p73 is increased in lung cancer, independent of *p53* gene alteration

**Y Tokuchi[1], T Hashimoto[1], Y Kobayashi[2], M Hayashi[1], K Nishida[2], S Hayashi[1], K Imai[1], K Nakachi[1], Y Ishikawa[3], K Nakagawa[4], Y Kawakami[5] and E Tsuchiya[1,2]**

[1]Saitama Cancer Center Research Institute, 818 Komuro, Ina, Saitama 362-0806, Japan; [2]Department of Clinical Pathology, Saitama Cancer Center Hospital, 818 Kumuro, Ina, Saitama 362-0806, Japan; Departments of [3]Pathology and [4]Respiratory Surgery, Cancer Institute, 1-37-1 Kami-Ikebukuro, Toshimaku, Tokyo 170-8455, Japan; [5]First Department of Medicine, School of Medicine, Hokkaido University, Kita 15 Nishi 7, Sapporo, 060-8636, Japan

**Summary** *p73* gene, a new *p53* homologue, has been identified: it supposedly acts as tumour suppressor gene in neuroblastoma. To clarify whether *p73* might be involved in lung carcinogenesis, we examined *p73* expression in resected lung cancer and paired normal lung in 60 cases using semi-quantitative reverse transcription-polymerase chain reaction (RT-PCR). We also examined *p73* gene status in three representative cases using Southern blot, and *p53* gene alteration in 49 cases using PCR-single-strand conformation polymorphism (PCR-SSCP) and direct sequence. In 87% of the cases (52/60) *p73* expression in tumour was more than twice as high as that in paired normal lung tissues, and the difference between *p73* expression in tumour and normal lung tissue was significant ($P < 0.0001$). However, Southern blot analysis revealed that none of the cases showed *p73* gene amplification. Compared with clinicopathological characteristics, *p73* expression correlates significantly with histological differences and age of patient, independently ($P < 0.05$). Concerning *p53* gene status, 43% (21/49) showed *p53* gene alteration, but there was no correlation between *p73* overexpression and *p53* gene alteration. Our results suggest that need for further functional analysis of the role of *p73* in lung carcinogenesis.

**Keywords:** lung cancer; *p73*; 1p36; *p53*; tumour suppressor gene

*p73*, a new candidate tumour suppressor gene, was recently identified at 1p36, the short arm of chromosome 1, by Kaghad et al (1997). The homology between *p73* and *p53* is extensive within most conserved *p53* domains (Zambetti et al, 1993; Ko et al, 1996; Kaghad et al, 1997). Wild *p53* works as a so-called security guard to induce cell cycle arrest or apoptosis in response to cellular stresses on DNA damage (Livingstone et al, 1992; Lowe et al, 1993; Dickman, 1997), and loss or inactivation of *p53* is thought to contribute to the development of 50% of all human cancers (Levine, 1997). Therefore, according to the known homology between *p53* and *p73*, *p73* is also expected to work as a tumour suppressor gene. Deletions of 1p36 are common in neuroblastomas, and extremely low level of *p73* mRNA have been found in the majority of neuroblastoma cell lines (Caron et al, 1995; Kaghard et al, 1997), showing that, at least in neuroblastoma, *p73* apparently works as a tumour supprressor gene in spite of the lack of definitive evidence.

However, very recently Mai et al (1998) reported that *p73* might be overexpressed in lung cancer tissues in comparison with normal lung. Furthermore, *p73* genomic mutation has not been found even after intensive research (Mai et al, 1998; Nomoto et al, 1998; Takahashi et al, 1998). Accordingly, it is questionable whether *p73* works as a tumour suppressor gene in lung cancer. To verify the role of *p73* in lung carcinogenesis, we measured the amount of *p73* mRNA expression in 86 primary lung cancer tissues and paired normal lung tissues by semi-quantitative reverse transcription-polymerase chain reaction (RT-PCR). We then analysed the relationship between the amount of *p73* expression and clinicopathological characteristics. Moreover, we examined for *p53* gene alteration to clarify the relationship between *p73* mRNA expression and *p53* gene status.

*Received 3 Sepember 1998*
*Revised 21 January 1999*
*Accepted 27 January 1999*

*Correspondence to:* E Tsuchiya

### MATERIALS AND METHODS

### Materials and histological classification

Surgically resected tumours and paired corresponding normal tissues were obtained from 86 patients with primary lung cancers at the Cancer Institute Hospital (Tokyo, Japan), from 1990 to 1993. All samples were quickly frozen in liquid nitrogen and stored at –80°C until RNA was extracted. RNA was estimated by β-actin mRNA expression amplified with RT-PCR, and we excluded 26 cases because of the poor quality of the extracted RNA. Distribution of histological types of the 60 lung cancers were: 34 adenocarcinomas, 19 squamous cell carcinomas, three large-cell carcinomas, two small-cell carcinomas and two adenosquamous carcinomas (World Health Organization, 1981) (Table 1). The median age of the 60 patients was 61 years (range 44–80 years). Forty-five of the 60 patients were men, and all 15 female patients had adenocarcinoma. The stage of each tumour was determined according to the *TNM Classification of Malignant Tumours* defined by the International Union Against Cancer (UICC, 1992): 19 stage I, 11 stage II, 21 stage IIIA, eight stage IIIB and one stage IV. To evaluate the consumption of cigarettes smoked before lung cancer diagnosis, a smoking index was used:

**Table 1** The relationship among characteristics of the patients, the amount of *p73* expression and *p53* gene status

| No. | Sex | Age | Histology[a] | p-Stage | Smoking index[b] | p73 in normal tissues[c] | p73 in tumour | p53 gene status[d] | Exon | Codon | Base change |
|---|---|---|---|---|---|---|---|---|---|---|---|
| 48 | M | 72 | Ad | II | 750 | 0.95 | 6.41 | ND | | | |
| 52 | M | 65 | Sq | IIIA | 900 | 1.78 | 27.88 | ND | | | |
| 54 | M | 70 | Sq | IIIB | 1250 | 2.54 | 6.14 | Mutant | 5 | 149–175 | deletion 74b |
| 55 | M | 59 | AS | I | 740 | 1.43 | 10.81 | ND | | | |
| 57 | M | 66 | L | IIIB | 1600 | 0.30 | 1.69 | ND | | | |
| 60 | M | 68 | Sq | IIIA | 940 | 0.34 | 8.14 | Wild | | | |
| 62 | M | 80 | Sq | II | 840 | 1.35 | 20.08 | Wild | | | |
| 63 | M | 76 | Sm | IIIA | 1250 | 4.08 | 58.06 | ND | | | |
| 64 | M | 54 | Sq | IIIB | 1350 | 1.04 | 3.93 | Wild | | | |
| 65 | M | 60 | Ad | IIIA | 800 | 0.28 | 0.84 | Wild | | | |
| 66 | W | 51 | Ad | IIIB | 0 | 0.41 | 0.42 | Mutant | 7 | 245 | GGC-AGC |
| 68 | W | 44 | Ad | IIIB | 0 | 0.69 | 1.58 | Wild | | | |
| 69 | M | 58 | Ad | IIIA | 1600 | 0.78 | 3.97 | Mutant | 8 | 273 | CGT-TGT |
| 70 | M | 59 | Sq | IIIA | 1200 | 0.72 | 9.62 | Mutant | 5 | 175 | CGC-CAC |
| 85 | M | 58 | Sq | IIIA | 1330 | 3.31 | 1.45 | ND | | | |
| 87 | M | 65 | Sq | I | 760 | 6.94 | 10.14 | Mutant | 7 | 244 | GGC-TGC |
| 91 | M | 65 | Ad | I | 2280 | 0.03 | 1.47 | Wild | | | |
| 93 | W | 61 | Ad | I | 310 | 0.42 | 2.44 | Wild | | | |
| 94 | M | 78 | Sq | I | 1020 | 0.78 | 29.20 | Wild | | | |
| 97 | M | 54 | Ad | IIIA | 960 | 0.22 | 0.48 | Mutant | 6 | 198 | GAA-TAA |
| 98 | M | 67 | Sm | IIIA | 940 | 0.05 | 1.88 | ND | | | |
| 99 | M | 70 | Sq | I | 1520 | 0.37 | 5.85 | Wild | | | |
| 101 | W | 68 | Ad | IIIA | 75 | 2.47 | 12.32 | Mutant | 7 | 242 | TGC-TAC |
| 102 | M | 53 | Sq | I | 234 | 0.24 | 2.12 | Wild | | | |
| 106 | M | 51 | AS | IIIA | 0 | 0.30 | 2.05 | ND | | | |
| 111 | M | 54 | Ad | I | 540 | 3.00 | 13.26 | Wild | | | |
| 114 | W | 77 | Ad | I | 0 | 0.70 | 2.72 | Wild | | | |
| 117 | W | 59 | Ad | IIIA | 0 | 1.51 | 5.33 | Wild | | | |
| 121 | M | 64 | Ad | II | 1320 | 0.38 | 3.32 | Wild | | | |
| 122 | W | 74 | Ad | IIIA | 240 | 0.88 | 3.33 | Mutant | 6 | 209 | AGA-TGA |
| 126 | M | 66 | L | IIIA | 460 | 0.53 | 4.63 | ND | | | |
| 127 | M | 44 | Ad | I | 340 | 0.06 | 0.66 | Wild | | | |
| 130 | W | 55 | Ad | I | 0 | 0.10 | 0.06 | ND | | | |
| 135 | M | 49 | Sq | IIIA | 300 | 0.46 | 4.02 | Mutant | 6 | 196 | CGA-CCA |
| 136 | M | 50 | Ad | I | 1600 | 0.30 | 6.51 | Wild | | | |
| 137 | M | 68 | Ad | IIIA | 0 | 0.11 | 0.35 | Wild | | | |
| 141 | M | 59 | Sq | I | 780 | 0.53 | 1.45 | Wild | | | |
| 142 | M | 70 | Ad | I | 561 | 0.55 | 2.79 | Wild | | | |
| 143 | M | 54 | Ad | IIIA | 1440 | 3.18 | 12.21 | Wild | | | |
| 146 | M | 76 | Sq | II | 1250 | 1.51 | 15.88 | Mutant | 5 | 144 | CAG-CCG |
| 147 | W | 68 | Ad | I | 0 | 0.40 | 0.19 | Wild | | | |
| 150 | M | 63 | Ad | IV | 600 | 0.17 | 0.54 | Wild | | | |
| 153 | W | 50 | Ad | II | 0 | 1.00 | 1.06 | Wild | | | |
| 157 | M | 49 | Ad | II | 20 | 1.21 | 2.16 | Mutant | 7 | 238 | TGT-AGT |
| 159 | M | 51 | Sq | II | 1240 | 0.12 | 5.64 | Mutant | 10 | 5 junction | agAT-tgAT |
| 160 | M | 50 | Ad | IIIB | 2310 | 0.62 | 3.23 | Mutant | 6 | 189 | CCC-G_C |
| 168 | W | 51 | Ad | II | 0 | 0.88 | 3.62 | Wild | | | |
| 174 | M | 72 | Ad | I | 510 | 2.00 | 2.59 | Mutant | 8 | 273 | CGT-CAT |
| 178 | M | 50 | Ad | I | 1750 | 0.61 | 4.89 | Wild | | | |
| 179 | M | 70 | Sq | IIIA | 1590 | 2.27 | 9.40 | Mutant | 6 | 195 | ATC-ACC |
| 188 | M | 53 | Sq | II | 1320 | 0.90 | 4.77 | Mutant | 8 | 271 | GAG-TAG |
| 193 | M | 68 | Sq | IIIA | 1440 | 1.33 | 12.47 | Mutant | 7 | 245 | GGC-GTC |
| 196 | M | 61 | Ad | II | 3680 | 1.20 | 3.74 | Wild | | | |
| 200 | M | 71 | Sq | IIIB | 1020 | 1.56 | 5.65 | Mutant | 4 | 103 | TAC-TAG |
| 201 | W | 51 | Ad | I | 0 | 0.10 | 2.44 | Wild | | | |
| 202 | W | 70 | Ad | IIIA | 0 | 0.65 | 4.13 | Wild | | | |
| 203 | W | 67 | Ad | IIIB | 0 | 0.74 | 5.65 | Mutant | 5 | 132 | AAG-AGG |
| 205 | W | 49 | Ad | I | 0 | 0.44 | 1.39 | Mutant | 6 | 213 | CGA-TGA |
| 206 | M | 49 | L | II | 1015 | 2.18 | 15.74 | ND | | | |
| 208 | M | 71 | Ad | IIIA | 960 | 1.72 | 10.75 | Mutant | 5 | 157 | GTC-TTC |

[a]Ad, Adenocarcinoma; Sq, Squamous cell carcinoma; Sm, Small cell carcinoma; L, Large cell carcinoma; AS, Adenosquamous carcinoma. [b]Smoking index is defined as cigarette consumption per day multiplied by smoking years. [c]*p73* expression is estimated by dividing by β-actin expression. [d]ND, not done.

*© 1999 Cancer Research Campaign*

cigarette consumption per day multiplied by smoking years. Heavy smokers were defined as those with smoking indices over 400.

### Preparation of RNA and expression of *p73* mRNA using RT-PCR

All tissue samples were frozen in liquid nitrogen immediately after surgery and subjected to isolation of RNA as previously reported (Tokuchi et al, 1999). RNAs were prepared from 0.1–0.2 g of human primary lung cancers and paired non-cancerous parenchyma according to the method of Chomczynski and Sacchi (1987), which was quantified using UV spectrometry at 260 nm. The RNAs (1.0 µg) in a 40 µl reaction volume were reverse transcribed to synthesize cDNA using Takara RNA PCR kit (Takara, Tokyo) with 2.5-µM random hexamers at 42°C. The oligonucleotides used in PCR amplification were as follows: P1 (sense strand in exons 4 and 5 of the *p73* gene), GAC GTA CTC CCC GCT CTT GA; P2 (antisense strand in exon 7 of the *p73* gene), TGG CTC ATA GGG CAC CAC GA; P3 (sense strand in exon 7 of the *p73* gene), ATT CAC CAC CAT CCT GTA CA; P4 (antisense strand in exon 9 of the *p73* gene), GCT GCT GCT GCT GCC GAT AG; P5 (sense strand of the β-actin), CAA GAG ATG GCC ACG GCT GCT; P6 (antisense strand of the β-actin), TCC TTC TGC ATC CTG TCG GCA. PCR amplification of the *p73* cDNA was performed using 1 µl of the cDNA, 0.2-µM primers P1 and P2 in 25-µl mixtures consisting of 1 U *Taq* DNA polymerase (Boehringer Mannheim), 0.05 MegaBequerel (MBq) of [α-$^{32}$P]dCTP, 10 mM Tris–HCI (pH 8.3), 50 mM potassium chloride, 1.5 mM magnesium chloride and 40 µM deoxynucleotide triphosphates (dATP, dTTP, dGTP and dCTP). The PCR reaction comprised 35 cycles with denaturing at 95°C for 30 s, and annealing and extension at 72°C for 20 s in each cycle, using a GeneAmp PCR System 9600 (Perkin-Elmer Corp.). Ten microlitres of the amplified product was subjected to 5% polyacrylamide gel electrophoresis, and the radioactivity was evaluated with Bio-Image analyser BAS2000 (Fuji Film, Tokyo). The PCR product size using P1 and P2 is 301 bases; using P3 and P4, 439 bases. No expected PCR products were observed when RT was not performed. To confirm the results with P1 and P2, an additional PCR using P3 and P4 was also performed: it comprised 40 cycles using a GeneAmp PCR System 9600 (Perkin-Elmer Corp.) in the buffer described above, with denaturing at 95°C for 30 s, and annealing and extension at 70°C for 1 min in each cycle.

As a control for RNA quality, separate portions of cDNA were amplified using primers specific for human β-actin. PCR was performed using 1 µl of cDNA, 1 U *Taq* DNA polymerase (Boehringer Mannheim), 0.5 µM P5 and P6, 0.05 MBq of [α-$^{32}$P]-dCTP and 0.15 mM magnesium chloride in 25-µl incubation buffers. The reaction (95°C and 63°C for 30 s, and 72°C for 1 min) was performed for 21 cycles.

To quantify the amount of PCR products of *p73* (using P1 and P2) and β-actin, 18 matched samples of lung tumours and normal lung tissues were processed simultaneously, and the radioactivity was determined with BAS2000 (Fuji Film, Tokyo, Japan). The amount of PCR product of each sample was expressed as a value relative to the average radioactivity of 18 normal lungs in each assay. Each PCR and electrophoresis procedure was repeated twice, after which we calculated the average *p73* and β-actin expression for each sample. Finally, we arrived at the relative *p73*:β-actin ratio, calculated by dividing the average amount of *p73* by that of β-actin, for each sample.

### *p73* genomic-Southern blot hybridization

Genomic DNAs from tumours and paired normal lung tissues of three representatives cases were extracted using standard methods as previously reported (Tsuchiya et al, 1992; Tokuchi et al, 1999). After each 10-µg DNA were completely digested with *Hin*dIII, DNAs were subjected to 0.8% agarose gel electrophoresis. Using vacuum blotter (Bio-Rad), DNA samples were rapidly transferred to the nylon membrane (Hybond-N; Amersham Japan, Tokyo, Japan) in 10 × standard saline citrate (SSC), and then cross-linked under UV light. RT-PCR products amplified with P1 and P2 primers, which contained exon 5, 6 and a part of exon 7 of *p73* coding sequence, were cut from the gel and purified using 0.45-µm centrifugal filter (Millipore), phenol–chloroform extraction and subsequent ethanol precipitation. After this, the purified DNAs were radiolabelled with [α-$^{32}$P]dCTP and multiprime DNA labelling systems kit (RPN. 1601Y, Amersham). Southern blot hybridization was performed at 59°C in 5 × SSC, 10 × Denhard's solution, 10 mM EDTA, 200 µg ml$^{-1}$ salmon sperm DNA and 1% sodium dodecyl sulphate (Sambrook et al, 1989). After sufficient washing, the radioactivity was evaluated with Bio-Image analyser BAS2000 (Fuji Film, Tokyo, Japan).

### *p53* mutation analysis and DNA sequencing

In 49 of the 53 patients with adenocarcinoma or squamous cell carcinoma, genomic DNAs were obtained and examined for *p53* alterations (exons 4–8 and 10) using PCR-single-strand conformation polymorphism (PCR-SSCP) (Kishimoto et al, 1992). Sequences of oligonucleotides used in PCR were as follows: exon 4 of *p53*, the sense primer, 5′-ACC TGG TCC TCT GAC TGC TCT TTT CA and the antisense primer, 5′-CCA GGC ATT GAA GTC TCA TGG AAG C; exon 5, 5′-TCT GTT CAC TTG TGC CCT GA and 5′-GCC AGA CCT AAG AGC AAT CA; exon 6, 5′-GCT GGG GCT GGA GAG ACG AC and 5′-GAC AAC CAC CCT TAA CCC CT; exon 7, 5′-CTT GCC ACA GGT CTC CCC AA and 5′-GGT CAG CGG CAA GCA GAG GC; expn 8, 5′-TTA AAT GGG ACA GGT AGG AC and 5′-GAT AAA AGT GAA TCT GAG GCA T; exon 10, 5′-TAT ACT TAC TTC TCC CCC TCC TCT and 5′-ATG AGA ATG GAA TCC TAT GGC TTT. The 5′-end of each primer was labelled with fluorescence: sense primer, 6-carboxyfluorescein, and antisense primer, 4,7,2′,7′-tetrachloro-6-carboxyfluorescein (Japan Bio Service Corp., Asaka, Japan). Genomic DNAs were extracted from 49 tumours and paired normal lung tissues (Tokuchi et al, 1999), and subjected to PCR reaction mixture containing 50 ng genomic DNA, 10 pM each pair of primers, 0.2 mM deoxynucleotide triphosphates (dATP, dTTP, dGTP and dCTP), 1.0 U *Taq* DNA polymerase (Boehringer Mannheim), 10 mM Tris–HCl (pH 8.3), 50 mM potassium chloride and 1.5 mM magnesium chloride. After initial denaturation (for 2 min at 94°C), 30 cycles PCR were carried out as follows: 94°C for 30 s, adequate annealing temperature for 30 s (exon 4, 69°C; exon 5, 53°C; exon 6, 63°C; exon 7, 64°C; exon 8, 59°C; and exon 10, 63°C) and 72°C for 30 s in a GeneAmp PCR System 9600 (Perkin-Elmer Corp.). PCR products were denatured at 98°C for 5 min, applied to 4% non-denaturing polyacrylamide gel with 10% glycerol, and electrophoresed at 22°C using ABI PRISM™ 377 (Perkin-Elmer Corp.). SSCP data were processed by GeneScan Analysis 2.0.2 computer software (Perkin-Elmer Corp.). Where genomic DNAs extracted from tumours showed different SSCP patterns from corresponding normal lung tissues, both genomic

© 1999 Cancer Research Campaign

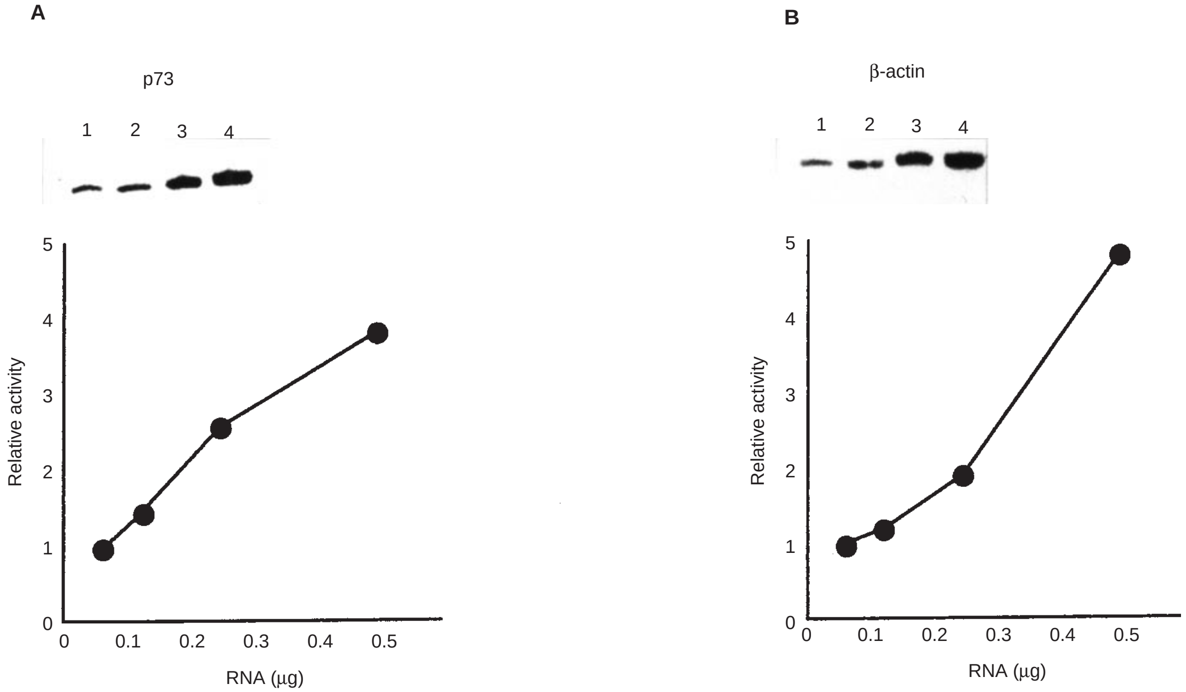

**Figure 1** Demonstration of relative radioactivity of *p73* (**A**) and β-actin (**B**) mRNA RT-PCR products. Various amounts of RNA of the tumour (case No. 101) in 20-μl reaction buffer were reverse-transcribed (lane 1, 0.0625 μg; lane 2, 0.125 μg; lane 3, 0.25 μg; lane 4, 0.5 μg), and each 1 μl cDNA was amplified with radiolabelled dCTP by PCR with P1 and P2, and with P5 and P6, as described in Materials and Methods. The dots on the graphs are the means of duplicate examinations

DNAs were amplified with the primers in the presence of [α-$^{32}$P]dCTP to elute the shifted DNA fragments for sequence analysis. After PCR in the same cycle condition, PCR products were electrophoresed in a 5% non-denaturing polyacrylamide gel with 10% glycerol at the most suitable temperature (exon 4, 10°C; exon 5–7 and 10, 25°C; exon 8, 15°C). After the gel was vacuum-dried and exposed to X-ray film, both normal and abnormal DNA fragments were eluted from the dried gel. Next, eluted DNAs were reamplified by PCR using the same primers, and the reamplified DNAs were sequenced using the dRhodamine terminator cycle sequencing kit (Applied Biosystems Inc.) and ABI PRISM™ 377 (Perkin-Elmer Corp.).

### Statistical analysis

All data were analysed using the Statistical Package for Social Sciences (SPSS), and a comparison of relative *p73*:β-actin expression ratios in tumours and in normal lung tissues was carried out using the Mann–Whitney *U*-test. The association between relative *p73*:β-actin expression ratio and clinicopathological parameters (age, sex, smoking habits, histological types and pathological staging) was estimated in 53 adenocarcinomas and squamous cell carcinomas, using the Mann–Whitney *U*-test and the Spearman rank correlation method. When any parameters significantly correlated with relative *p73* expression, we used the partial correlation analysis to exclude the interaction among these parameters. Linear regression was used to test the association between *p73*:β-actin expression ratio and clinical characteristics; the optimal regression model was chosen using the backward step-wise selection of variables. Moreover, relative *p73*:β-actin expression ratio was compared with *p53* gene alteration in 49 of the above 53 adenocarcinomas and squamous cell carcinomas using the Mann–Whitney *U*-test. All *P*-values < 0.05 were considered significant.

### RESULTS

To check the linear relationship between amount of RNA and radiolabelled PCR products in the semi-quantitative RT-PCR method, various amounts of total RNA were processed by RT-PCR amplification. The mean value of the duplicate *p73* PCR products amplified by using P1 and P2 increased dose-dependently at 35 cycles of amplification (Figure 1A), and a similar relationship between β-actin PCR products and amounts of RNA was also observed (Figure 1B).

Figure 2 shows the representative *p73* RT-PCR products from paired normal lungs and tumours, in which increased *p73* expression was observed in tumours compared with normal lung tissues. Use of another set of primers, P3 and P4, confirmed the results of PCR using P1 and P2 (data not shown). In 87% of the cases (52/60), the relative *p73*:β-actin ratio in tumours is more than twice as high as that in normal tissues, but the reverse is found in only 5% of the cases (3/60, No 85, 130 and 147 in Table 1).

Distribution of the relative *p53*:β-actin ratio both in normal lung tissues and in tumours in shown in Figure 3. The expression of *p73* mRNA in lung tumours is distributed significantly higher than that in normal lungs ($P < 0.0001$, Mann–Whitney *U*-test); the means amounts of p73 mRNA in lung tumours and normal tissues were 6.76 and 1.10 respectively.

In order to examine whether the overexpression of p73 exclusively observed in lung tumours was due to the gene amplification, Southern blot analysis was performed using genomic DNAs.

 © 1999 Cancer Research Campaign

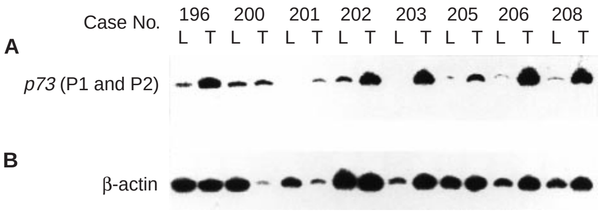

**Figure 2** Representative autoradiogram of RT-PCR products of *p73* (**A**) and β-actin (**B**) mRNA in lung cancer and normal tissue. T, lung cancer tissue; L, paired normal lung tissue. (**A**) p73 expression amplified with P1 and P2 as described in Materials and Methods; (**B**) amplified with P5 and P6

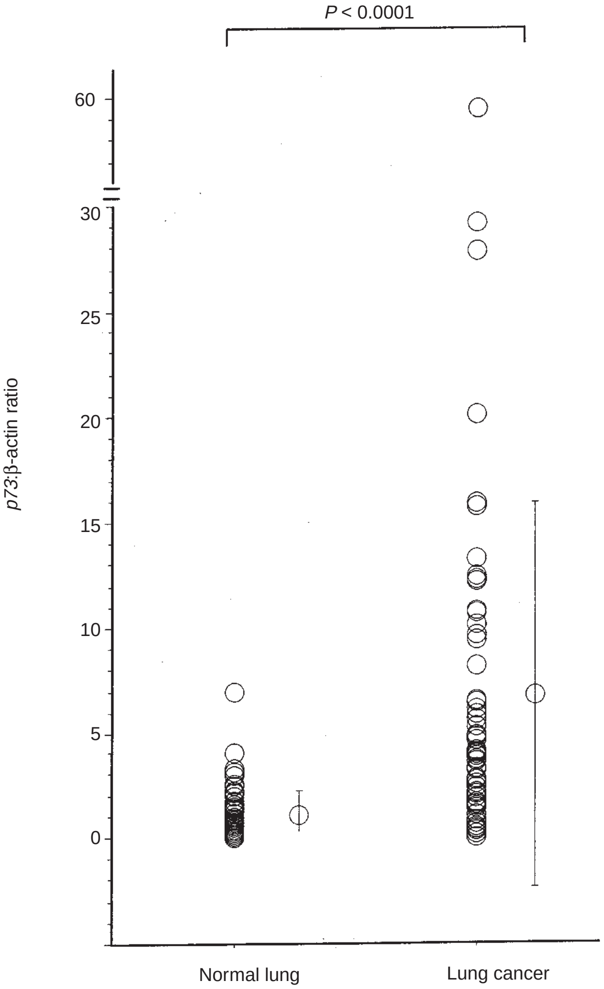

**Figure 3** Distribution of the relative *p73*; β-actin expression ratio in 60 paired normal lung tissues and lung tumours. Circles with bars indicate the mean value and the standard deviation

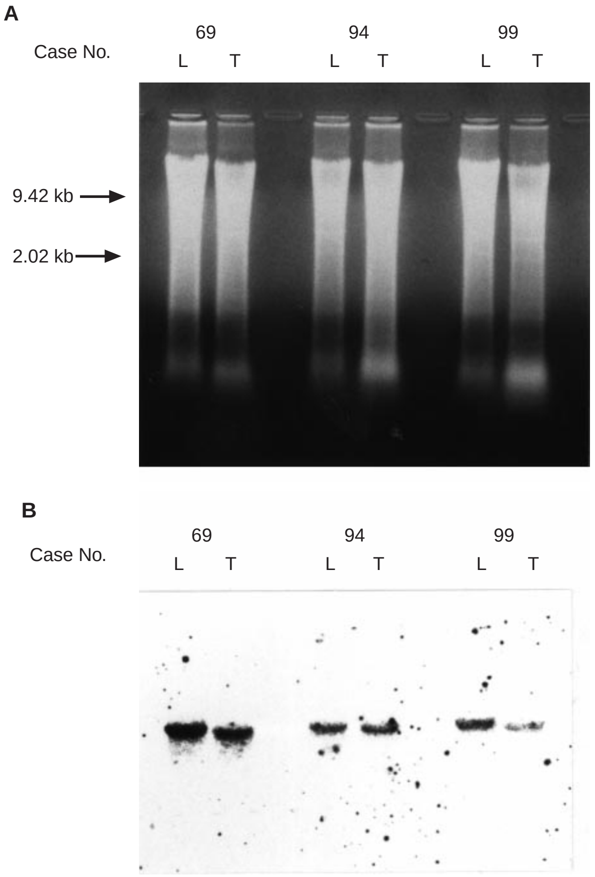

**Figure 4** Southern blot analysis of *p73* gene using genomic DNA extracted from three representative cases in which *p73* mRNA in lung cancer tissues was expressed stronger than in normal lung tissues. (**A**) Ethidium bromide stained gel electrophoresis after digestion of 10-µg genomic DNA with *Hin*dIII; (**B**) Southern blot hybridization with the labelled *p73* cDNA probe (containing exons 5, 6 and a part of exon 7 of *p73*), which was amplified by RT-PCR with P1 and P2 as described in Materials and Methods. T, lung cancer tissue; L, paired normal lung tissue

Genomic DNAs were extracted from three representative cases in which *p73* mRNA expression was markedly higher in tumours than in normal tissues: none of these cases showed the amplification of *p73* gene (Figure 4), thus eliminating amplification as the cause of *p73* overexpression in these three cases.

Next, we analysed the relationship between the relative *p73*:β-actin ratio and the clinicopatholigical features in 53 lung adenocarcinomas and squamous cell carcinomas, using the Mann–Whitney *U*-test and the Spearman rank correlation. The relative *p73*:β-actin ratios in tumours were found to significantly correlate with differences of histology (adenocarcinoma or squamous cell carcinoma), age, sex and smoking index (Tables 2 and 3). However, we could not ignore inter-relationships within these parameters, so we applied the partial correlation analysis. We found that only differences in histology and age significantly correlated with the relative *p73*:β-actin ratio independently of other factors (Table 4). This result was reconfirmed by the optimal multivariate linear regression model with the backward step-wise selection of variables: the final model obtained after the steps included age and histological types only as significant and independent variables.

Concerning the comparison of *p73* expression in normal lung tissues and clinicopathological parameters, the relative *p73*:β-actin expression ratio in normal tissues is significantly associated with the age of the patients and *p73* expression in paired tumours ($P<0.05$ respectively, the Spearman rank correlation). Accordingly, the relative *p73*:β-actin expression ratio in normal tissues also increases with age, although it is far lower than in tumours.

We also examined mutational status of *p53* gene in 92% of the cases (49/53) with adenocarcinomas or squamous cell carcinomas

**Table 2** The relationship between the amount of *p73* expression and clinicopathological features in 53 adenocarcinomas and squamous cell carcinomas

| Clinical features | No. of cases | *p73* : β-actin ratio[a] | *P*-value[b] |
|---|---|---|---|
| Age | | | |
| <61 | 26 | 3.74 ± 3.47 | 0.018 |
| ≥61 | 27 | 7.91 ± 7.69 | |
| Sex | | | |
| Men | 38 | 6.95 ± 6.93 | 0.023 |
| Women | 15 | 3.11 ± 3.08 | |
| Histology | | | |
| Adenocarcinoma | 34 | 3.73 ± 3.60 | 0.001 |
| Squamous cell carcinoma | 19 | 9.68 ± 8.20 | |
| p-Stage | | | |
| I | 18 | 5.01 ± 6.97 | 0.15 |
| ≥II | 35 | 6.30 ± 6.00 | |
| Smoking index | | | |
| <400 | 20 | 2.80 ± 2.79 | 0.001 |
| ≥400 | 33 | 7.72 ± 7.12 | |
| *p53* gene status | | | |
| Wild | 28 | 5.17 ± 6.49 | 0.11 |
| Mutant | 21 | 6.19 ± 4.34 | |

[a]Mean ± s.d. [b]Mann–Whitney *U*-test.

by PCR-SSCP, in exons 4–8 and exon 10, and subsequent direct sequencing: we found that 43% (21/49) had mutant *p53* (Table 1), much the same frequency as in previous reports (Kishimoto et al, 1992). The mean levels of *p73* expression in 21 mutant *p53* cases and 28 wild *p53* cases are 6.19 and 5.17 respectively, and there was no statistically significant correlation between *p53* gene alteration and *p73* expression ($P$ = 0.11, Table 2). Moreover, even when we divided the subjects into two groups by histological difference, no association was found between *p53* gene alteration and *p73* expression.

### DISCUSSION

The *p53* gene and its protein product have been the centre of intensive cancer studies since more than 50% of human cancers contain this gene abnormality (Levine, 1997). Because *p73* closely resembles *p53* in transactivation (29% identity with *p53* amino acids), DNA binding (63% identity with *p53*), and *p53* oligomerization (38% identity with *p53*) domains, *p73* is thought to play an important role in the development and/or progression of various types of human cancers (Dickman, 1997; Kaghard et al, 1997; Oren, 1997). In fact, *p73* can enhance levels of endogenous p21/Waf1 protein, the representative target of *p53*, and *p73* can also inhibit cell growth by inducing apoptosis in a *p53*-like manner (Jost et al, 1997; Kaghard et al, 1997).

In the present study we clearly showed that levels of *p73* mRNA expression is higher in tumours than in normal tissues, as two previous papers dealing with only a few samples had reported (Mai et al, 1998; Takahashi et al, 1998). In four reports the authors failed to find *p73* genomic mutation despite intensive search (Kaghard et al, 1997; Mai et al, 1998; Nomoto et al, 1998; Takahashi et al, 1998). Judging from these results, it was possible that wild-type *p73*, not mutant *p73*, might be overexpressed exclusively in tumours. Furthermore, our results revealed that *p73* overexpression was not due to gene amplification but, in all probability, to induction of transcription or to stabilization of *p73* mRNA. Moreover, we showed that *p73* overexpression is independent of *p53* gene alteration.

There are two possible explanations of the relationship between *p73* overexpression and lung carcinogenesis. The first is that *p73* may work as a security guard (Dickman, 1997): we found no direct relationship between *p53* mutation and *p73* overexpression in the present study, but the cell cycle in cancer cells is generally faster than in normal cells regardless of *p53* gene status, so *p73* might work to arrest the cell cycle as a tumour suppressor gene. The

**Table 3** Spearman rank correlation coefficients for *p73* : β-actin ratio in tumours and clinicopathological characteristics in 53 adenocarcinoma and squamous cell carcinomas

| | Sex | Age | Histology | Smoking index | p-Stage | p73/β-actin ratio |
|---|---|---|---|---|---|---|
| Sex | 1.0 | | | | | |
| Age | 0.10 | 1.0 | | | | |
| Histology | 0.47[a] | 0.21 | 1.0 | | | |
| Smoking index | 0.75[a] | 0.09 | 0.38[a] | 1.0 | | |
| p-Stage | 0.02 | 0.02 | 0.09 | 0.08 | 1.0 | |
| *p73*:β-actin ratio | 0.32[b] | 0.40[a] | 0.46[b] | 0.30[a] | 0.10 | 1.0 |

[a]$P < 0.01$, [b]$P < 0.05$.

**Table 4** Partial correlation analysis for *p73* : β-actin ratio in tumours and clinicopathological characteristics in 53 adenocarcinoma and squamous cell carcinomas

| | Partial correlation coefficients | Variables under control |
|---|---|---|
| Sex | 0.05 | Age, Histology, Smoking index, p-Stage |
| Age | 0.39[a] | Sex, Histology, Smoking index, p-Stage |
| Histology | 0.34[b] | Sex, Age, Smoking index, p-Stage |
| Smoking Index | 0.04 | Sex, Age, Histology, p-Stage |
| p-Stage | –0.05 | Sex, Age, Histology, Smoking index |

[a]$P < 0.01$; [b]$P < 0.05$

 *© 1999 Cancer Research Campaign*

second possibility is that *p73* might act as an oncogene in up-regulation of cell growth, as Mai et al (1998) pointed out. Kaghad et al (1997) reported that p73 protein is neither stabilized nor activated by DNA damage, including damage from UV radiation or actinomycin D, so it is clearly different from *p53*. Because the expressions of *p73* and *p53* are induced in different manners, it is possible that *p73* may have a role different from that of *p53* in lung cancer carcinogenesis. And since, in the present study, *p73* overexpression was observed exclusively in lung tumour, *p73* may possibly work as an oncogene in lung carcinogenesis.

In the present study, our results suggest that *p73* overexpression in tumours correlates with histological type and age of patients. Furthermore, *p73* expression in normal lung tissues also correlates with age of patients, although *p73* expression in normal lung tissues is much lower than that in tumours. Accordingly, *p73* expression might be associated with the changes accompanying aging of the host or differences of histological construction in tumors. These observation will provide some clue as to why *p73* is overexpressed in lung tumour.

In conclusion, *p73* expression in lung tumours is obviously greater than its expression in normal tissues, independent of *p53* gene alteration, indicating that the role of *p73* in lung carcinogenesis might be different from its role in neuroblastoma. Further study is needed of this possible new function of p73 protein.

### ACKNOWLEDGEMENTS

We thank Drs H Sugano (Cancer Institute) and T Kozu (Saitama Cancer Center) for their helpful advice. We also thank Drs S Tsuchiya and S Okumura at Cancer Institute Hospital, for kindly providing lung cancer samples. The technical assistance of T Yoshikawa and Y Yamaoka is gratefully acknowledged. This work is supported by Grant-in-Aid for Science Research from Ministry of Education, Science, Sports and Culture of Japan; Ministry of Health and Welfare of Japan; Selectively Applied and Developed Research from Saitama Prefecture and the Smoking Research Foundation.

### REFERENCES

Caron H, Peter M, van Sluis P, Speleman F, de Kraker J, Laureys G, Michon J, Brugieres L, Voute PA, Westerveld A, Slater R, Delattre O and Versteeg R (1995) Evidence for two tumor suppressor loci on chromosomal bands 1p35-36 involved in neuroblastoma: one probably imprinted, another associated with N-myc amplification. *Hum Mol Genet* **4**: 535–539

Chomczynski P and Sacchi N (1987) Single-step methods of isolation by acid guanidium thiocyanate–phenol–chloroform extraction. *Anal Biochem* **162**: 156–159

Dickman S (1997) First p53 relative may be a new tumor suppressor. *Science* **277**: 1605–1606

Framer G, Bargonetti J, Zhu H, Freiedman P, Prywes R and Prives C (1992) Wild-type p53 activates transcription in vitro. *Nature* **358**: 83–86

Jost CA, Martin MC and Kaelin WG Jr (1997) p73 is a human p53-related protein that can induce apoptosis. *Nature* **389**: 191–194

Kaghard M, Bonnet H, Yang A, Creancier L, Biscan J-C, Valent A, Minty A, Chalon P, Lelias J-M, Dumont X, Ferrara P, McKeon F and Caput D (1997) Monoallelically expressed gene related to p53 at 1p36, a region frequently deleted in neuroblastoma and other human cancers. *Cell* **90**: 809–819

Kastan MB, Zhan Q, El-Deiry WS, Carrier F, Jacks T, Walsh WV, Plunkette BS, Vorgelstein B and Fornance AJ Jr (1992) A mammalian cell cycle checkpoint pathway utilizing p53 and GADD45 is defective in atalaxia-telangiectasia. *Cell* **71**: 587–597

Kern SE, Pietenpol JA, Thiagalingam S, Seymour A, Kinzler KW and Vogelstein B (1992) Oncogenic forms of p53 inhibit p53-regulated gene expression. *Science* **256**: 827–830

Kishimoto Y, Murakami Y, Shiraishi M, Hayashi K and Sekiya T (1992) Aberrations of the p53 tumor suppressor gene in human non-small cell carcinomas of the lung. *Cancer Res* **52**: 4799–4804

Ko LJ and Privies C (1996) p53: puzzle and paradigm. *Genes Dev* **10**: 1054–1072

Levine AJ (1997) p53, the cellular gate keeper for growth and division. *Cell* **88**: 323–331

Livingstone LR, White A, Sprouse J, Livanos E, Jacks T and Tisty TD (1992) Altered cell cycle arrest and gene amplification potential accompany loss of wild-type p53. *Cell* **70**: 923–935

Lowe SW, Schmitt EM, Smith SW, Osborne BA and Jacks T (1993) p53 is required for radiation-induced apoptosis in mouse thymocytes. *Nature* **362**: 847–849

Mai M, Yokomizo A, Qian C, Yang P, Tindall DJ, Smith DI and Liu W (1998) Activation of p73 silent allele in lung cancer. *Cancer Res* **58**: 2347–2349

Nomoto S, Haruki N, Kondo M, Konishi H, Takahashi T, Takahashi T and Takahashi T (1998) Search for mutations and examination of allelic expression imbalance of the p73 gene at 1p36.33 in human lung cancers. *Cancer Res* **58**: 1380–1383.

Oren M (1997) Lonely no more: p53 finds its kin in a tumor suppressor haven. *Cell* **90**: 829–832.

Sambrook J, Fritsch EF and Maniatis T (1989) *Molecular Cloning: A Laboratory Manual*. Cold Spring Harbor, New York: Cold Spring Harbor Laboratory.

Takahashi H, Ichimiya S, Nimura Y, Watanabe M, Furusato M, Wakui S, Yatani R, Aizawa S and Nakagawara A (1998) Mutation, allelotyping, and transcription analysis of the p73 gene in prostatic carcinoma. *Cancer Res* **58**: 2076–2077.

Tokuchi Y, Kobayashi Y, Hayashi S, Hayashi M, Tanimoto K, Hashimoto T, Nishida K, Ishikawa Y, Nakagawa K, Satoh Y, Yamamoto M and Tsuchiya E (1999) Abnormal FHIT transcripts found in both lung cancer and normal lung tissue. *Genes Chromosomes Cancer* **24**: 105–111

Tsuchiya E, Nakamura Y, Weng S-Y, Nakagawa K, Tsuchiya S, Sugano H and Kitagawa T (1992) Allelotype of non-small cell lung carcinoma: comparison between loss of heterozygosity in squamous cell carcinoma and adenocarcinoma. *Cancer Res* **52**: 2478–2481.

UICC (International Union Against Cancer) (1992) *TNM Classification of Malignant Tumours*, 4th Edn, Revision 2. Berlin: Springer-Verlag

World Health Organization (1981) *Histological Typing of Lung Tumours*. Geneva: World Health Organization.

Zambetti GP and Levine AJ (1993) A comparison of the biological activities of wild-type and mutant p53. *FASEB J* **7**: 855–865.

© 1999 Cancer Research Campaign
